# Spinal disorders mimicking infection

**DOI:** 10.1186/s13244-021-01103-5

**Published:** 2021-12-04

**Authors:** Sana Boudabbous, Emilie Nicodème Paulin, Bénédicte Marie Anne Delattre, Marion Hamard, Maria Isabel Vargas

**Affiliations:** 1 Division of Radiology, Department of Diagnosis, Geneva University Hospitals, Rue Gabrielle-Perret-Gentil 4, 1211 Geneva 14, Switzerland; 2 Faculty of Medicine of the Geneva University, Geneva, Switzerland; 3 Division of Radiology, Medical Imaging Department, Hospital of Neuchatel, Neuchâtel, Switzerland; 4 Division of Neuroradiology, Diagnostic Department, University Hospitals of Geneva, Geneva, Switzerland

**Keywords:** Spondylodiscitis, Magnetic resonance imaging, Inflammation, Degenerative

## Abstract

Spinal infections are very commonly encountered by radiologists in their routine clinical practice. In case of typical MRI features, the diagnosis is relatively easy to interpret, all the more so if the clinical and laboratory findings are in agreement with the radiological findings. In many cases, the radiologist is able to make the right diagnosis, thereby avoiding a disco-vertebral biopsy, which is technically challenging and associated with a risk of negative results. However, several diseases mimic similar patterns, such as degenerative changes (Modic) and crystal-induced discopathy. Differentiation between these diagnoses relies on imaging changes in endplate contours as well as in disc signal. This review sought to illustrate the imaging pattern of spinal diseases mimicking an infection and to define characteristic MRI and CT patterns allowing to distinguish between these different disco-vertebral disorders. The contribution of advanced techniques, such as DWI and dual-energy CT (DECT) is also discussed.

## Key points

- Spondylodiscitis in elderly patients is challenging in presence of inflammatory Modic changes.
- Features of Anteroposterior inflammatory spine is suggestive of metabolic disease.
- Approach of destructive spondylodiscitis should be integrated in clinical context.
- CT is complementary to MRI to assess osseous changes and detect calcifications.
- 5-Disco-vertebral biopsy could be avoided by systematic analysis of vertebral endplates signal.

## Introduction

Spondylodiscitis represents 2–7% of all osteomyelitis cases, with an annual incidence of 1/100,000 to 1/250,000 [1–3]. Disco-vertebral biopsy is usually not mandatory if a pathogenic agent is detected by blood culture, yet the detection rate is low (24%) [4]. Symptoms are initially non-specific and the diagnosis is frequently delayed for 2 to 6 weeks [5]. Neurological symptoms, such as leg weakness, sensory deficit, or radiculopathy, are present in one-third of cases [6]. CT guided biopsy for identifying the pathogenic agent is mainly indicated in atypical and mycobacterial infections in order to choose the appropriate treatment. However, this technique is invasive and at times challenging, notably in kyphotic patients. In addition, it may be associated with complications, particularly in case of difficult access, high risk of bleeding, or high risk of injuries to not-targeted organs R1 Point 1 even if recent studies report high accuracy and safety in expert hands [7–9]. Not to mention that the success rate is low-to-moderate, ranging from 28.1 to 57.1% [10, 11]. Moreover, pathogenic agent detection is lower in sclerotic areas [1]. C-reactive protein (CRP) levels higher than 50 mg/L as well as the presence of a paravertebral phlegmon, soft tissue abscess, or epidural abscess are correlated with a higher rate of positive biopsy results [1]. In this review, the most common and reliable MRI and CT features of metabolic and degenerative disorders mimicking spondylodiscitis will be described and illustrated. The imaging pattern of these differential diagnoses will also be discussed.

### Technical imaging

MRI is the technique of choice in the acute phase of spondylodiscitis, with a sensitivity of up to 92% (increased to 95% when using a contrast agent) and a specificity of up to 96% [12]. No difference has been reported according to the MRI field strength (1.5 or 3 T (T)) based on morphological T1 and T2WI. STIR is usually used to better detect edema. Sagittal planes are preferred and have to be completed by axial planes for better visualization and delineation of phlegmon or abscesses. Fat-saturation T1WI following contrast agent administration are additionally used to highlight the inflammatory process and detect abscesses. DWI has been used more recently mainly to distinguish between spondylodiscitis and degenerative disorders as well as spinal tumor involvement, with ADC values of inflammatory endplates determined at 1.5 and 3 T [13]. R2 Point 1 Hence, ADC values for spinal infection oscillated between 1.27 and 1.52 × 10^−3^ mm^2^/s depending on b-value [12, 14, 15].

However, the choice of b-value is an important point to consider when analyzing these results. Several authors [14–17] concluded that correct choice of b-value is mandatory for interpreting this sequence (sensitive to both perfusion and cellular density when low and only to cellular density when high), the solution being to use a relatively high b-value without compromising the signal-to-noise ratio. An example of protocol with detailed parameters as performed in our institution at 1.5 and 3 T is provided in Table 1.

**Table 1:** Technical MRI protocol of spondylodiscitis at 3 Tesla

Sequence	Acquisition time	TE/TR (ms)	TI (ms)	FOV (mm)	Matrix	Slice thickness (mm)/gap	Voxel size	Parallel imaging acceleration factor (GRAPPA)
t2 tse sag (3 stacks)	3 min 39 × 3	102/3500		270	384	4/10%	0.4 × 0.4	2
t1 tse sag (3 stacks)	1 min 59 × 3	9/450		270	320	4/10%	0.8 × 0.8	2
t2 tirm sag (3 stacks)	3 min 51 × 3	36/3700	220	270	320	4/10%	0.8 × 0.8	2
t2 tirm cor (psoas)	4 min 35	36/3700	220	340	84	4/10%	0.9 × 0.9	2
Diffusion resolve**	5 min 09	60/95/2630*		220	128	4/10%	1.7 × 1.7	2
t1 tse dixon sag with Gd (3 stacks)	3 min 03 × 3	11/500		270	320	4/10%	0.8 × 0.8	2
t1 tse fatsat tra with Gd	2 min 41	11/717		190	256	4/10%	0.7 × 0.7	2

TSE, TurboSpinEcho; Sag, sagittal; Cor, coronal; Fatsat, fat saturation; Tra, transverse; Gd, gadolinium; min, minute; mm, millimetre; ms, millisecond; TE, echo time; TR, repetition time; TI, inversion time

*Resolve sequence has 2 TE

**Sag 4b values (b0, 300, 600, 800)

CT is used to guide percutaneous biopsy, permitting to identify the causative microbiological agent and to exclude malignancy and mimicking diagnoses [1]. In addition, 3D reconstructions are useful for surgeons when spinal fixation is planned [18].

Dual-energy CT (DECT) is a technique enabling tissue quantification based on energy dependence at different X-ray spectra [19]. It is used to assess bone infiltration, such as in multiple myeloma, degenerative diseases like disc herniation [20], and gout. DECT-evidence of urate deposition is considered as a criterion for gout, with this imaging modality having a high sensitivity and specificity (87% and 84%, respectively) [21], but strong data are lacking for the spine. Radionuclide imaging is a useful alternative to MRI when the latter is contraindicated, 18F-fluorodeoxyglucose (^18^F-FDG) being the radiotracer of choice [22, 23]. R1 Point 3^18^F-FDG is a reliable technique to monitor response to treatment with high sensitivity and specificity [24, 25]. X-ray is no longer used in the diagnostic setting due the delay (2 to 8 weeks) in the appearance of radiographic features [26] and the risk of missing the diagnosis in the degenerative spine [27]. Nowadays, this modality, performed in loading position, is reserved to preoperative assessment of spinal deformation.

### Imaging pattern of infectious spondylodiscitis

The typical MRI pattern in the acute stage consists of inflammatory endplates that appear hyperintense on T2 and STIR and hypointense on T1, along with disc hypersignal (in mirror) [22]. Hyperintensity on T2 and STIR sequences typically involves the entire disc, with enhancement after contrast agent administration and loss of intranuclear cleft. This feature is also known as the “hot disc sign” [28]. Although regarded as controversial in some studies [29], it is highly suggestive of spondylodiscitis (more than 90% of infection cases). Paraspinal or epidural abscesses are complications of spondylodiscitis and they appear in high signal intensity on T2WI and STIR sequences, and in low signal intensity with annular or heterogeneous enhancement on T1WI. Erosions are a late feature and indicate a subacute progress. On DWI, the mean ADC of infectious spondylodiscitis was lower (1.27 ± 0.38 × 10^−3^ mm^2^/s) than for the control group, but higher than in patients with degenerative discitis [9]. For other authors, the ADC cut-off value for spinal infection was 1.52 × 10^−3^ mm^2^/s when using a b-value of 800 s/mm^2^ [14–17]. Patel et al. demonstrated that R2 point 2 the preservation of linear regions of high signal situated within the adjacent vertebral bodies, described as the “claw sign”, can be used to distinguish between degenerative changes in the spine (Modic 1), in which case the claw sign is present, and spondylodiscitis, in which case MRI shows heterogeneous amorphous hyperintensity or absent crab’s claw sign [30]. Sequences allowing generating several contrasts as well as quantifying T1 and T2 relaxation times, such as synthetic imaging or fingerprinting, are promising to differentiate between spondylodiscitis and metabolic or degenerative diseases using quantitative imaging [31]. Spondylodiscitis CT features depend on the osteitis stage. Lytic defects (erosions) of the endplates without sclerotic margins indicate an earlier stage [32]. Besides, a sclerotic pattern of endplates is correlated with a higher risk of negative biopsy [32]. CT remains the technique of choice to assess spinal calcifications [20].

R1 Point 2 On the other hand, differentiation between pyogenic and tuberculosis spondylodiscitis based only on imaging remains challenging. The presence of an intact meningo-vertebral ligament (posterior midvertebral septum) [33], thoracic involvement, subligamentous extension, skip lesions, importance of abscesses and kyphotic deformity [34] as well as disc space sparing and vertebral body collapse [35] are suggestive of tuberculosis.

Finally, imaging criteria could be integrated in a scoring system for the diagnosis and to monitor progression following treatment [36].

### Imaging findings of diseases mimicking spinal infection on MRI

The typical MRI characteristics are summarized in Table 2, which is provided as a guide to differentiate diseases mimicking infectious spondylodiscitis according to signal intensity changes and additional X-ray and CT characteristic features.

**Table 2:** Common MRI features in spondylodiscitis and mimicking diseases

Entity	Localization	Endplates	Disc	Soft tissues	Associated features	Pattern XR/CT
Spondylodiscitis	Lumbar spine	Blurred endplates Diffuse hypersignal STIR in mirror Amorphous enhancement	Hypersignal (hot sign disc)	Heterogeneous paraspinalepidural abscesses		Endplate defects without sclerosis
Modic 1	Lumbar spine	Irregular contours but intact endplates Ratio of edema on T1 confined to subchondral bone Mixed pattern with inflammation and fat Pseudo-sparing, ghost sign after contrast Crab’s claw sign on DWI and lower ADC	Absence of hypersignal on T2 and STIR (unless vacuum phenomenon)	Slight infiltration, no abscesses	Several levels	Irregular and pseudo-cystic endplates
Crystal diseases	Cervical > lumbar > thoracic	Erosive and hyperintensity Enhancement	Hyperintensity	Inflammation of soft tissues	Tophi and pyrophosphate deposits on hyposignal T1 and T2 Involvement of facet joints	Dense masses (tophi or CCPD) in other disc or facet joints with erosive pattern DECT + + + (gout)
Spondyloarthropathy	Thoracolumbar junction Mobile segment	Hypersignal with enhancement on hemispheric shaped pattern, Romanus lesion	Possible hypersignal on T2 and STIR, transdiscal fracture	No abscesses, no epiduritis	Several levels Chest wall and sacroiliac involvement Posterior extension of fracture	Syndesmophytosis Bone ankylosis Kyphotic deformity
Neuropathic spine	Thoracolumbarlumbosacral	Advanced destruction	Advanced discitis, vacuum disc	Variable signal depending on amount of debris and edema fluid collection (excessive motion)	Involvement of facet joints, osseous debris, spondylolisthesis, joint disorganization	Hypertrophic osteophytosisspinal deformity
SAPHO	Thoracolumbar	Erosions, sclerosis, and bony bridging Semicircular pattern of hyperintensity on STIR enhancement Hypointensity in the chronic phase	Disc narrowing Rarely hypersignal on T2 and STIR and T1 with contrast	Contiguous involvement Thickening and inflammation of spinal ligaments Posterior facet joints and spinous process Sterno-clavicular, sacro-iliac		
Compression fracture	-	Edema fracture line	Normal	Absence of epidural and soft tissue abscesses		
Acute Schmorl node	Thoracolumbar	High signal T2 and STIR, enhancement, concentric ring feature, one endplate	No disc abnormality			

## Modic 1 degenerative changes of the endplate

**Fig. 1:**
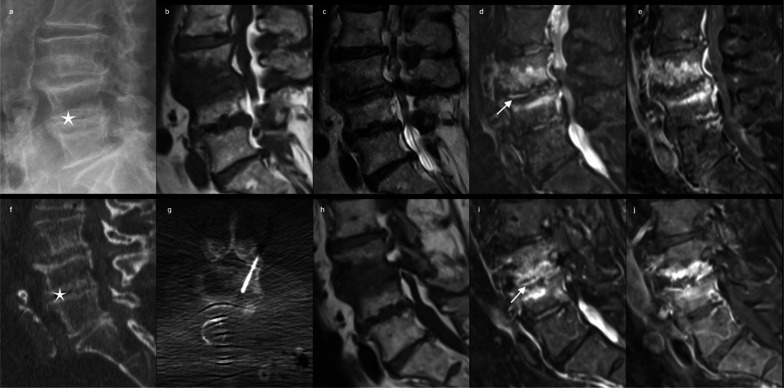
68-year-old male with increasing back pain referred for pathologic confirmation and treatment of spondylodiscitis. Sagittal XR (**a**), Sagittal T1 (**b**), T2 (**c**), STIR (**d**) and T1 Fatsat after gadolinium R1 point 5 (**e**) confirm edema-type enhancing marrow signal abnormalities as well as disc hyperintensity (arrow), and narrowed L3–L4 space without disc enhancement. Following sagittal CT (**f**) R1 point 6 confirms the absence of endplates destruction and that the hypersignal on the underlying disc (L4–L5) corresponds to the vacuum phenomenon (star), biopsy performed under CT guidance (**g**) was negative. At 3 months, MRI control on sagittal T1 (**h**), STIR (**i**) and after contrast (**j**) shows stability of disco-vertebral features (arrow)

Degenerative spinal disorders in the inflammatory stage (Modic 1) can mimic infection particularly due to an overlapping clinical presentation (acute spinal pain and inflammatory laboratory findings). The vertebral endplate changes are related to inflammation-mediated active disc degeneration [37, 38]. On the other hand, the increased vascularity seen in Modic 1 predisposes to non-specific, low-grade bacterial infections. For vascular and biomechanical reasons, Modic lesions and spondylodiscitis have a similar location, R2 point 3 predominantly affect the lumbar levels [39]. Conventional CT is sensitive to endplate destruction, with a very good specificity. It appears irregular and can be associated with subchondral cysts, but edema cannot be visualized in early stages [40]. There are no available data in the literature regarding assessment of Modic lesions by DECT. On MRI, bone marrow edema appears hypointense on T1WI and hyperintense on T2WI and STIR, with a pattern of claw sign on DWI. Abnormal disc signal intensity and contrast enhancement are non-specific patterns (Fig. 1). Moreover, endplate erosions are seen in infections and in degenerative processes due to osteochondrosis. Thus, morphological differentiation is important and can be assessed using several features. Schwartz-Nemec et al. [41] showed that endplate contours, irregular but intact in Modic 1 and blurred in spondylodiscitis, is the most accurate sign to distinguish between Modic 1 and spondylodiscitis. Secondarily, the edema extent in the vertebral body and the T1 signal ratio (between edematous area and unaffected bone marrow) are considered as important features to distinguish the two entities [41]. A careful edema analysis reveals a mixed pattern of inflammation and fat in most cases of Modic lesions, contrary to spondylodiscitis [41]. This underlines the central role of the T1WI sequence to make the distinction between Modic lesions and spondylodiscitis with high accuracy. Pseudo-sparing [42], which refers to increased visibility of endplate contours, and the “ghost sign” [43], which refers to the reappearance of cortical bone on T1WI sequence after contrast injection, have also been described as being suggestive of infection. The extension of edema beyond the subchondral bone on fat-suppressed fluid-sensitive MRI images is an additional feature suggestive of infection rather than Modic 1, but this finding has been subject of controversy due to an overlap between infection and degenerative disorders [44]. A thin hypointense marrow between the disc and bone edema on STIR sequence may be present and reflects sclerosis, which is suggestive of Modic 1 rather than infection [45]. Regarding the disc signal, an absence of hyperintensity on T2WI is critical to make the diagnosis of Modic 1 [46]. Nevertheless, disc hypersignal is not specific of spondylodiscitis and could be related to a vacuum phenomenon in Modic changes due to disc disruption [41]. Even if no paravertebral abscess is visible, a very slight infiltration can be noticed. Concerning DWI, results of studies comparing ADC values between normal, degenerative, and infectious diseases [47] demonstrated a lower ADC value (1.12 ± 0.22 × 10^−3^ mm^2^/s) in degenerative diseases [13]. Furthermore, the extension and shape of abnormalities in the vertebral body can be characteristic on DWI and the “claw sign” [30], are suggestive of Modic 1 in opposition to an amorphous increased signal on DWI, which has been suggested to be a good indicator of infection [30].

## Andersson lesion in ankylosing spondylitis

**Fig. 2:**
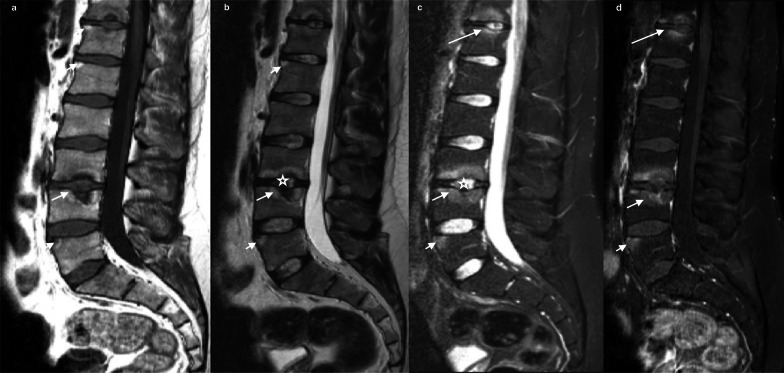
32-year-old male admitted for exacerbation of back pain and inflammatory markers. T1 (**a**), T2WI (**b**), and STIR- R1 point 7 (**c**) show inflammatory endplate changes at the L3–L4 level mimicking a Schmorl node (arrow). Note that nuclear cleft sign is preserved (star). Enhancement in T1 fat-suppressed sequence after contrast (**d**) without disc enhancement or inflammatory paraspinal tissue. The same pattern is seen in the D11–D12 disc space (long arrow), associated with Romanus feature R1 point 8 at L5 body margin (short arrow)

**Fig. 3:**
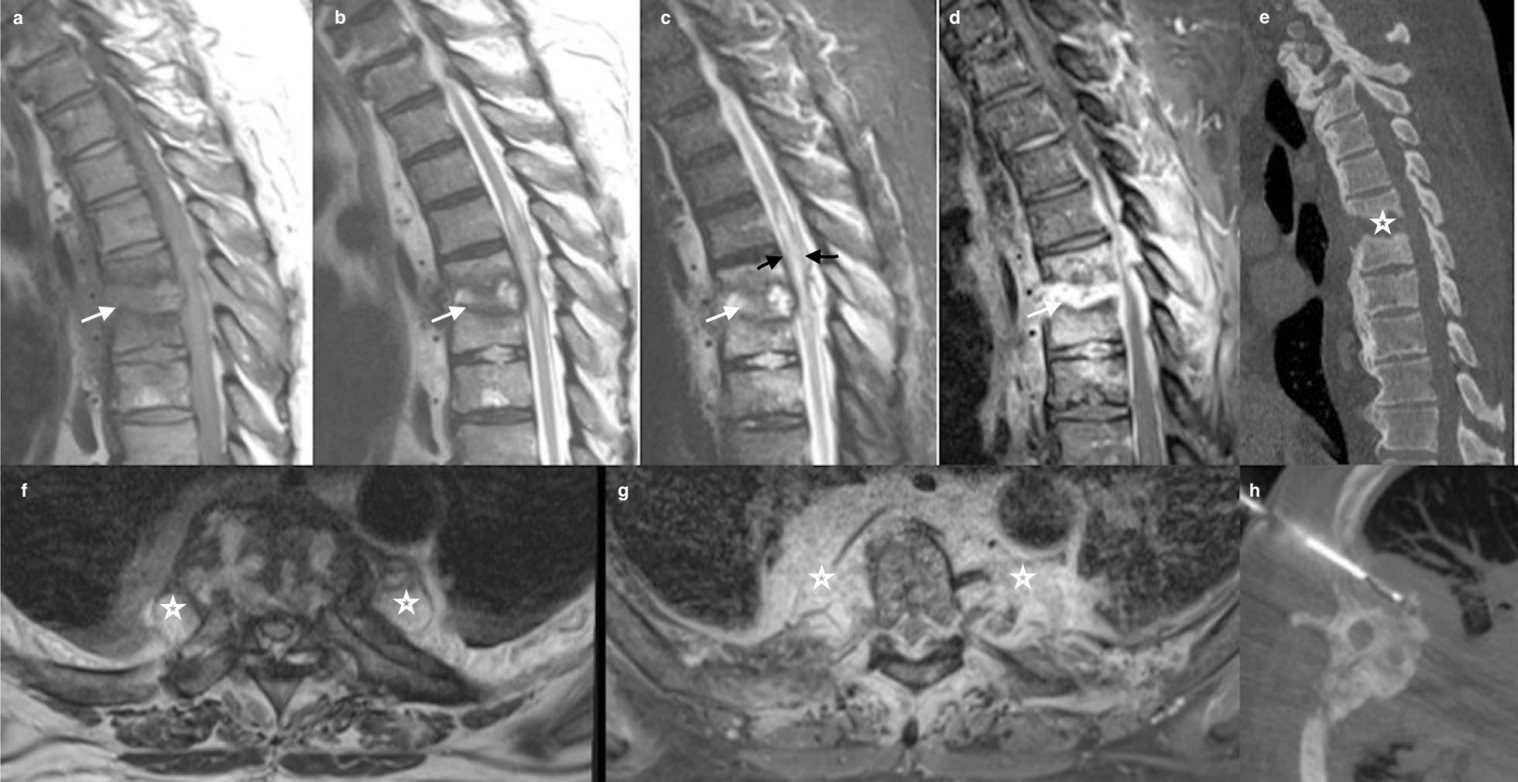
R1 point 9: 78-year-old male with sepsis and thoracic back pain with ankylosing spondylitis. MRI (sagittal T1 (**a**), T2 (**b**), STIR (**c**) and T1 Fat Sat Gadolinium (**d**)) shows T6-T7 disc hypersignal, enhanced after administration of contrast (arrow) and marked inflammatory surrounding tissue in axial T2 (**f**) and axial T1FS with contrast (**g**) (star). Note the elevated medullary signal intensity secondary to compression (black arrow). Reformatted CT (**e**) shows ankylosed spine and transdiscal fracture (star). Discal biopsy (**h**) was performed to exclude an infection R1 point 10. Andersson discitis was diagnosed

The Andersson lesion, which was first described by Andersson in 1937, is a rare but well-known destructive disco-vertebral complication of ankylosing spondylitis [48]. Though it is believed to be a late complication, many authors have reported it in the early stage of this rheumatoid disease. Its prevalence varies between 1.5 and 28% [49]. The disco-vertebral destruction is mediated by both inflammation and a micro-traumatic process, such as pseudarthrosis. Inflammation is accounted for by an acute enthesopathy [50], with an extension from anterior spondylitis to discs and endplates [51]. The micro-traumatic process, which is confirmed histologically by the presence of fibrosis and chondrodysplasia [52], occurs at the disc level [53]. Spondyloarthritis can affect one or several levels and predominates at the thoracolumbar junction [54–56]. Misdiagnosis with infectious discopathy, mainly in relation with tuberculosis in endemic areas, may lead to unnecessary investigations and costs [57]. Destructive and sclerotic changes on X-ray, CT, and MRI are frequent in chronic recurrent spondylitis and are known to be late features [53]. Discitis is possible, with narrowing and increased signal on T2WI and STIR, but no case of abscess or epiduritis has been reported in Andersson lesion. Endplate signal abnormalities appear as hypersignal on STIR and as hyposignal on T1WI, very often with a hemispheric-shaped pattern [53] (Fig. 2). While syndesmophytosis can be missed in these cases, it is highly suggestive of the disease and well detected by radiographs or CT, which additionally shows erosive and remodeled endplates with disc space narrowing [45]. Chest wall involvement and sacroiliac lesions are frequently associated [49]. MRI shows features of degenerative disease that mimic sclerotic Modic 3 [58]. Features of Romanus lesion are frequent, have to be detected in these cases, and also help to make the diagnosis [52]. Patients with spondyloarthropathy develop osteoporosis due to inflammation and kyphotic deformity (related to bone ankylosis) and they exhibit an increased risk of fractures at the thoracolumbar junction involving the “weak” segment, which is the disc. Hypertrophic nonunion is a well-known feature in this “mobile segment” and leads to pseudarthrosis mimicking an erosive disco-vertebral disease, yet sclerosis and fracture visualization in the posterior column permit diagnosis [59] (Fig. 3).

## Crystal-induced discopathy

**Fig. 4:**
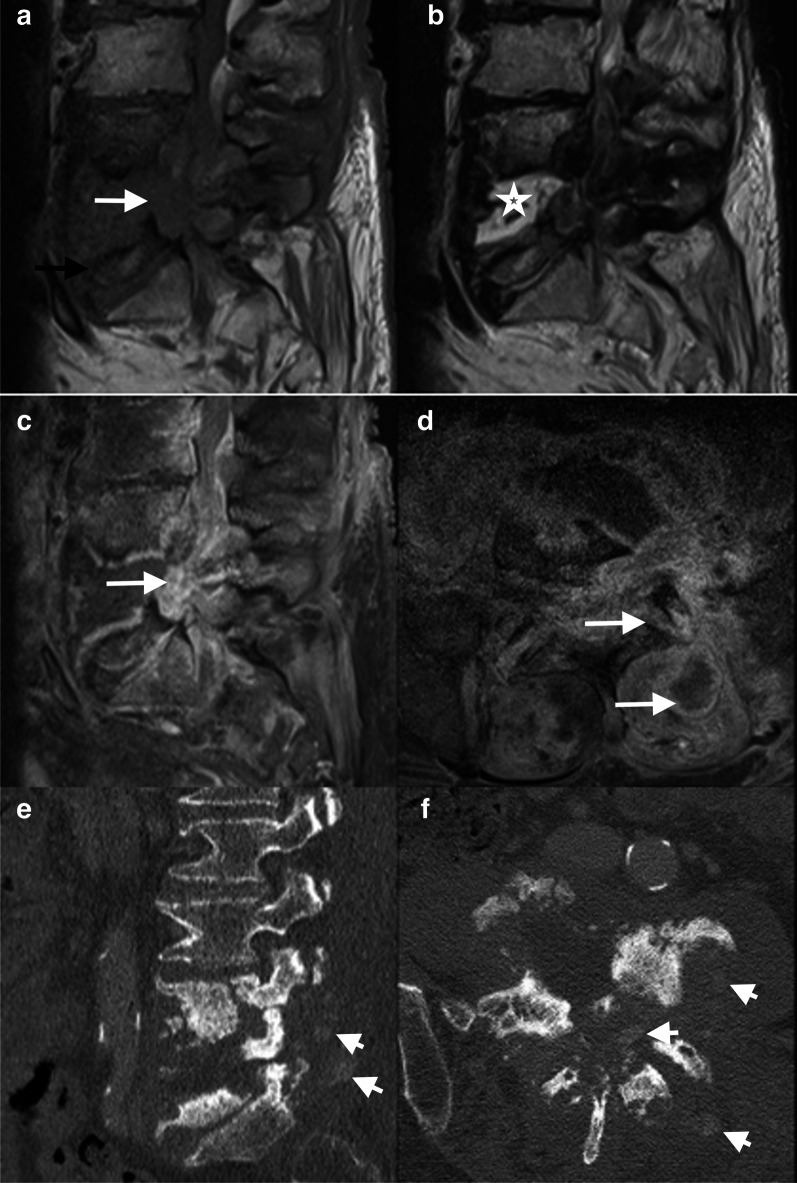
70-year-old male with chronic history of back pain, recently exacerbated, and inflammatory laboratory markers. Sagittal T1WI (**a**) shows hypointense discal changes in the L4–L5 space and epidural space (arrow). Bony erosion of endplates is also seen (black arrow) at the L5 level (**b**, **c**). Sagittal T2WI (**b**) shows hyperintense areas in the L4–L5 disc space (star). Contrast-enhanced sagittal and axial T1WI (**c**, **d**) show enhancement of disc space, epidural space, left facet joint, and paraspinal muscles (arrow). Non-enhancing foci correspond to crystal material (arrow). Axial and sagittal slices of CT (**e**, **f**) show in L4–L5, disc space erosions, sclerotic vertebral bodies, and confirm gout tophi in muscles (short arrow)

Crystal deposition in the disc may mimic osteomyelitis in case of acute inflammatory presentation. Pathogenically, crystals form in the pericellular matrix of cartilage, thereby inducing inflammation and catabolic effects on chondrocytes and synoviocytes and resulting in pericellular matrix destruction; besides, metabolic deposits may accelerate joint damage [60]. The real prevalence of these spinal disorders is unknown and probably underestimated [61, 62], mainly because of an increasing incidence in the elderly population [63, 64]. Only case reports are available in the literature [65, 66]. Crystal diseases predominate in males, with a wide range of age (median of 59 years for gout) [61]. In most cases, a prior history of crystal disease is known. However, the prevalence of presentation as “spondylodiscitis” has not been reported in the published series. While the cervical spine is usually affected, the lumbar spine may also be involved in rare cases; though rare, gout is the most frequent crystal-induced discopathy [63, 67, 68]. R1Point 4 Toprover et al. reported the largest series including 133 cases of spinal gout [61], which exhibits the clinical characteristics of arthritis [69]. Calcium pyrophosphate deposition (CPPD) disease causes an acute inflammation by mass effect [63, 67]. It predominates in the cervical spine (crowned dens syndrome), followed by the lumbar and rarely the thoracic spine [70]. In the majority of cases, disco-vertebral biopsies are negative and MRI features are misdiagnosed as an infection. MRI is the most used modality, being pathologic in more than 90% of cases, but without specific findings. An erosive pattern in adjacent vertebral plates is frequent, along with an increased signal in the disc and surrounding soft tissues. Tophi or CPPD appear hypo- or isointense compared to muscles on T1 and T2WI with peripheral enhancement [69, 71, 72] (Fig. 4). Hence, these signal abnormalities help to make the diagnosis, although signal may be hyperintense even on T1 or T2WI. Tophi or CPPD can usually be visualized on radiographs and especially on CT, yet not in all cases, and the most common findings are joint erosions associated to dense masses [73, 74]. Performing both a CT scan to detect crystal deposition at other disc levels or in posterior joints and to show sclerotic margins and surrounding dense masses in case of gout and an aspiration or biopsy is key to diagnosis. The recent development of spectral imaging, such as DECT, is promising to detect monosodium urate crystal deposition and thus allow differentiation between several materials [75, 76], without further need of percutaneous or surgical biopsies. The final diagnosis can be made using routine polarized light microscopy, which reveals negatively birefringent urate crystals in case of gout or calcium pyrophosphate dihydrate crystals in case of chondrocalcinosis (pseudo-gout). Crystal diseases with no neurological emergency can effectively be managed using a conservative treatment (anti-inflammatory prophylaxis and urate-lowering therapy).

## Neuropathic spinal arthropathy

**Fig. 5:**
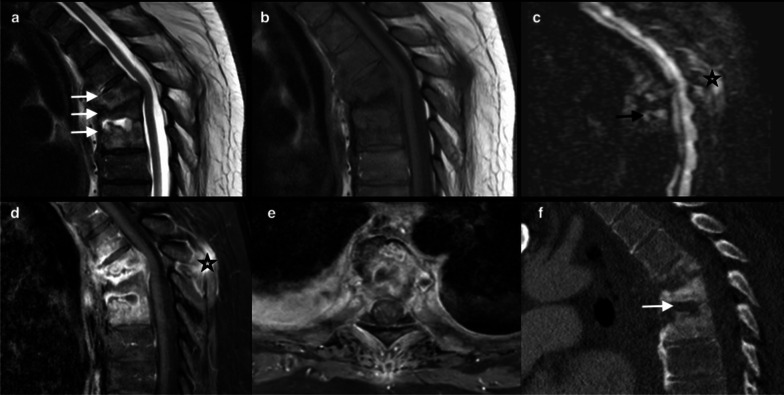
72-year old male with amyotrophic lateral sclerosis presenting with subacute back pain, fever and inflammation in laboratory findings. Sagittal T2 (**a**), T1 (**b**) WI, DWI (**c**) and T1 after contrast on sagittal (**d**) and axial planes (**e**) show multiple contiguous erosive disco-vertebral disease on dorsal spine with local deformity and advanced destruction (arrow). No diffusion restriction was noticed on DWI (black arrow). Note anterior and posterior involvement (star). Reformatted sagittal CT (**f**) shows bone sclerosis and subtle vacuum phenomena in the disc (arrow). R1 Point 11The final diagnosis was Charcot spine

Also known as Charcot spine, this condition is characterized by a rapid destruction of disco-vertebral and facet joints due to several neuropathic disorders [77]. The underlying mechanism is a loss of protective sensation and proprioceptive reflexes [78]. Delay in diagnosis is the rule, in addition to non-specific imaging features. Spinal cord traumatic injury is the most common underlying condition affecting the mobile segments above or below the stabilized one [79]. Hence, the thoraco-lumbar and lumbosacral segments are most commonly involved. The atrophic form of this disease is characterized by bone resorption and may mimic septic arthropathy. Thus, spondylodiscitis is the main differential diagnosis. MRI is best suited for differentiation using morphological and functional sequences [80]. MRI shows an advanced destruction of endplates and discs as well as an involvement of facet joints, which are rarely associated in spondylodiscitis [77]. Disco-vertebral destruction is usually accompanied by hypertrophic osteophytosis and spinal deformity [81]. Soft tissue masses are typically hypointense on T1WI and isointense to hyperintense on T2WI, depending on bone debris and variable amount of edema (Fig. 5). An associated fluid collection due to excessive motion is characteristic of this disease during the atrophic phase [78]. The main features allowing distinction between neuropathic spinal arthropathy and spondylodiscitis include vacuum disc phenomenon, osseous debris, spondylolisthesis, and joint disorganization [82, 83]. Of note, secondary infections have been observed in 17% of cases [84]. The imaging features of neuropathic spinal arthropathy are classically described as the six D’s, standing for: Distension (for soft tissue mass), Density (for sclerosis), Debris (for bone fragmentation), Disorganization (for joint dislocation), Destruction (for endplate and facet erosions), and Dislocation (for spondylolisthesis) [85].

### Synovitis, acne, pustulosis, hyperostosis, osteitis (SAPHO) syndrome

**Fig. 6:**
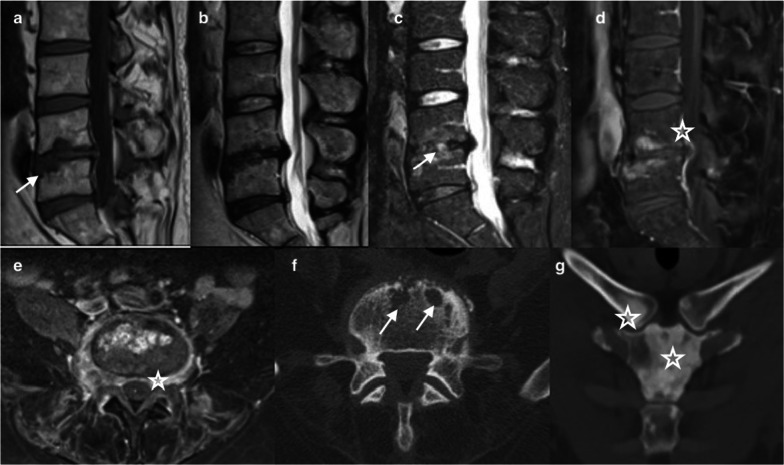
47-year-old male presenting with inflammatory back pain. Sagittal T1 (**a**) and R1 point 12 T2 (**b**)-WI show erosive L4-L5 endplates (arrow) associated on Sagittal STIR sequence (**c**) to a disc hypersignal (arrow) and on sagittal (**d**) and axial (**e**) T1 Fat Sat WI enhanced sequences to an enhancement of vertebral bodies and surrounding tissues as epidural fat space without abscess (star). Axial CT (**f**) on lumbar spine shows erosive pattern of vertebra (arrow) and Oblique reformatted CT (**g**) of anterior chest shows involvement with sclerotic lesions typical of SAPHO disease (star)

Spinal involvement is the second most frequent manifestation of the synovitis, acne, pustulosis, hyperostosis, osteitis (SAPHO) syndrome [86], with thoraco-lumbar segments being most commonly affected [87]. It is characterized by vertebral endplate erosions, subchondral sclerosis, and bony bridging. These abnormalities predominate on the anterior side of the vertebra, although posterior facet joints and spinous process are also frequently affected. Contiguous involvement is frequent, being observed in up to 89% of cases [88]. Abnormalities are described as having a semicircular pattern (Fig. 6). In the active phase, MRI shows the bone marrow edema as hypointense on T1WI and as hyperintense on T2, STIR, and T1WI following contrast injection. Moreover, it detects the surrounding soft tissue inflammation. Soft tissue mass is due to thickening and inflammation of spinal ligaments [89]. In cases of SAPHO syndrome, disc narrowing is frequent, but hypersignal and enhancement after contrast injection are very rare (11%) [88], yet they have been described by some authors [90]. These findings may mimic spondylodiscitis, while sterile spondylodiscitis has been described in 9 to 32% of SAPHO syndrome cases [91]. An association with hypointensities in the chronic phase and the involvement of other sites (e.g., sterno-clavicular and sacro-iliac findings) help to make the diagnosis. Whole-body MRI offers the possibility to explore all affected sites besides the spine [92].

## Miscellaneous

### Disc disorders associated with vertebral fractures

Vertebral compression fractures may mimic spondylodiscitis, mostly tuberculous spondylitis, if the endplate is poorly defined in the absence of an obvious trauma history [93]. MRI shows bone marrow edema and allows ruling out a discitis. The lack of epidural extension and paraspinal mass enables to make the diagnosis. Finally, vertebral fractures may complicate mycobacterial infections or pyogenic spondylitis at an early stage [94].

### Acute schmorl nodes

Schmorl nodes represent an extrusion of a disc through an endplate around the cartilaginous node. They manifest as high signal intensity on T2WI and STIR and low signal intensity on T1WI and contrast enhancement, with a correlation with pain in some cases. The concentric ring feature and involvement of an endplate without signal intensity abnormalities allow ruling out the diagnosis of spondylodiscitis [95].

## Conclusion

Although MRI features of disco-vertebral infections are usually quite typical, there are a variety of degenerative, post-traumatic, metabolic, and non-infectious inflammatory conditions that may present with similar features. Awareness of the differential diagnoses described above and knowledge of the specific pattern on MRI may help the radiologist to avoid these pitfalls and an unnecessary disco-vertebral biopsy.

## Data Availability

The datasets used and/or analyzed during the current study are available from the corresponding author on reasonable request.

## Abbreviations

^18^F-FDG: 18F-fluorodeoxyglucose
ADC: Apparent diffusion coefficient
CRP: C reactive protein
CT: Computed tomography
DECT: Dual-energy computed tomography
DWI: Diffusion weighted imaging
MRI: Magnetic resonance imaging
SAPHO: Synovitis, Acne, Pustulosis, Hyperostosis and Osteitis
SPD: Spondylodiscitis
STIR: Short tau inversion recovery
T: Tesla
